# Intestinal perforation on an incarcerated incisional hernia secondary to an ingested foreign body. Report of a rare case

**DOI:** 10.1093/jscr/rjab348

**Published:** 2021-08-14

**Authors:** Ricardo Vaz-Pereira, Cátia Ferreira, Ana Monteiro, Gonçalo Guidi, Daniela Martins, João Pinto-de-Sousa

**Affiliations:** Department of General Surgery, Centro Hospitalar De Trás-Os-Montes E Alto Douro, E.P.E, Av. Noruega, 5000-508 Vila Real, Portugal; Department of General Surgery, Centro Hospitalar De Trás-Os-Montes E Alto Douro, E.P.E, Av. Noruega, 5000-508 Vila Real, Portugal; Department of General Surgery, Centro Hospitalar De Trás-Os-Montes E Alto Douro, E.P.E, Av. Noruega, 5000-508 Vila Real, Portugal; Department of General Surgery, Centro Hospitalar De Trás-Os-Montes E Alto Douro, E.P.E, Av. Noruega, 5000-508 Vila Real, Portugal; Department of General Surgery, Centro Hospitalar De Trás-Os-Montes E Alto Douro, E.P.E, Av. Noruega, 5000-508 Vila Real, Portugal; Department of General Surgery, Centro Hospitalar De Trás-Os-Montes E Alto Douro, E.P.E, Av. Noruega, 5000-508 Vila Real, Portugal

## Abstract

Ingestion of foreign bodies (FBs) is common and rarely has consequences for the patient, but sometimes it can originate gastrointestinal perforation and lead to devastating consequences if unrecognized. Therefore, whenever present, bowel perforation demands immediate surgical treatment.

An 89-year-old woman with an incarcerated incisional hernia, whose imaging study was consistent with intestinal occlusion and perforation within the hernia sac was treated at our hospital. A segmental enterectomy and direct correction of the hernial defect were performed. A perforation in the mesenteric border due to a FB, which seemed to be a toothpick, was identified in the surgical specimen. Nine months after surgery, the patient was without complaints, with adequate healing, and without evidence of hernial recurrence.

To the best of our knowledge, this is the first case of intestinal perforation on an incarcerated incisional hernia, due to an ingested FB, reported in the literature.

## INTRODUCTION

The ingestion of foreign bodies (FB), whether incidentally or intentionally, is quite common. Most cases are accidental [1, 2], and indeed, in >75% of cases, they are spontaneously eliminated [3]. Common risk factors for ingesting FB are the use of dentures or dental prostheses, and most of them are bones, fishbones and toothpicks [1, 4]. Bowel perforation seems to be rarely observed, occurring in <1% of the situations [5], but whenever present they demand urgent treatment. To the best of our knowledge, no cases of bowel perforation, on an incarcerated incisional hernia, due to ingested FB, have been previously reported in the literature.

Herein, a rare case of contained intestinal perforation, within the sac of an incarcerated incisional hernia, due to ingested FB, which seemed to be a toothpick, is reported.

## CASE REPORT

**Figure 1:**
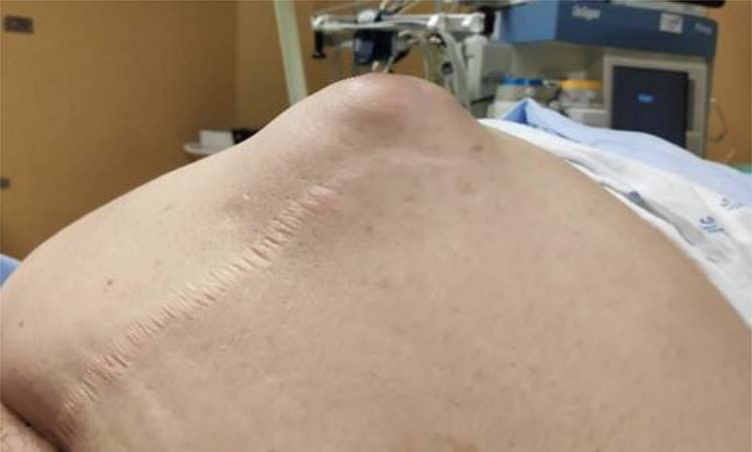
Incarcerated incisional hernia.

An 89-year-old woman, partially dependent, had a history of hypertension, type 2 diabetes mellitus, morbid obesity class III (body mass index 43 kg/m^2^), chronic obstructive pulmonary disease, obstructive sleep apnea syndrome and non-stratified heart failure was observed at our hospital. The patient was brought to the emergency department due to generalized abdominal pain, nausea and vomiting with 24 hours of evolution and complaining of no gas or feces emission for the last 48 hours. On physical examination, she evidenced signs of dehydration, blood pressure was 106/47 mmHg, heart rate 106 bpm and body temperature 37°C. A scar from a median umbilical and infraumbilical incision was present in her abdomen examination. A non-reducible umbilical hernia could be observed, with skin erythema, pain and guarding (Fig. 1).

**Figure 2:**
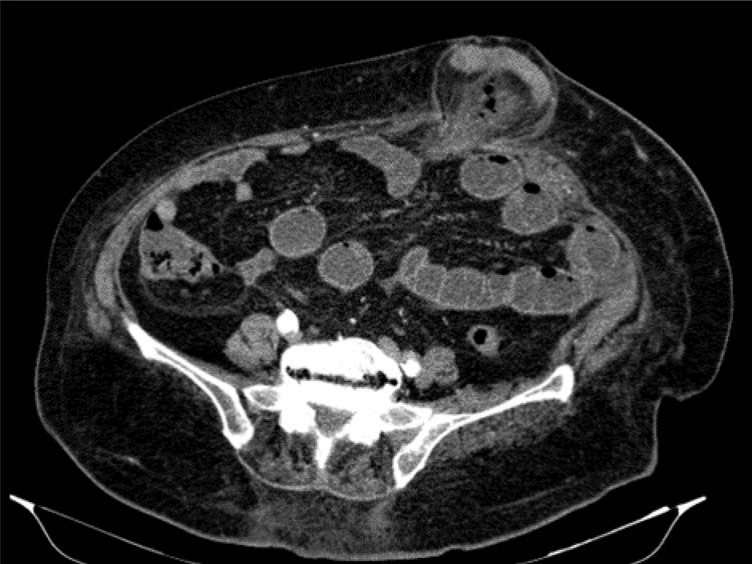
Abdominal CT: hernia content composed of intestinal loop and densification of the mesentery with gas bubbles.

**Figure 3:**
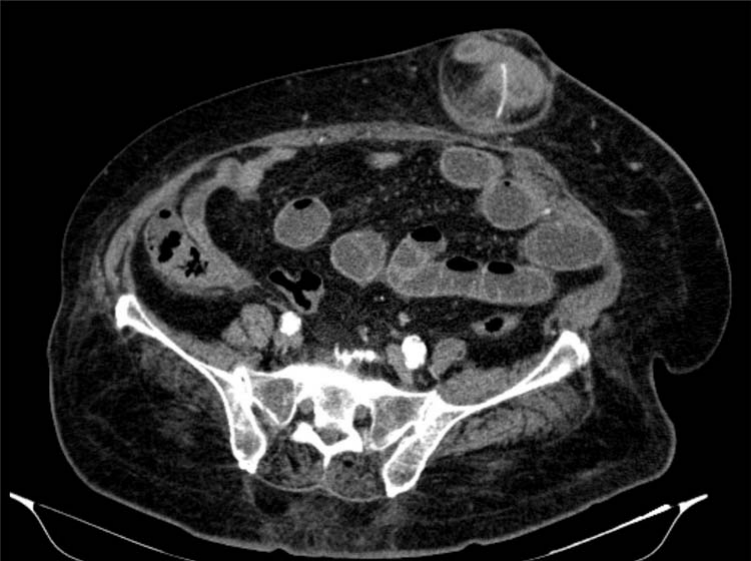
Abdominal CT: image of high-density and filiform morphology compatible with FB.

Fluid resuscitation measures, monitoring of urine output and pain control were initiated. Analytically, she had leukocytosis of 227 000/μl with neutrophilia 89%, elevated C-reactive protein 27.1 mg/dl and acute kidney injury with creatinine 1.8 mg/dl and urea 96 mg/dl. Arterial blood gas analysis showed no acidosis, respiratory failure or hyperlactatemia. The computed tomography (CT) scan identified an umbilical hernia containing a small bowel loop, evidenced densification of the hernia fat and some bubbles of extraluminal gas were also present (Fig. 2). An image of high-density and filiform morphology compatible with an FB (Fig. 3) was also identified. Images were consistent with dilation of the proximal intestinal loops, which suggested bowel occlusion.

**Figure 4:**
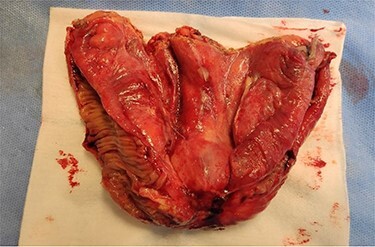
Open enterectomy specimen, in which marked thickening of the mesentery is identified.

**Figure 5:**
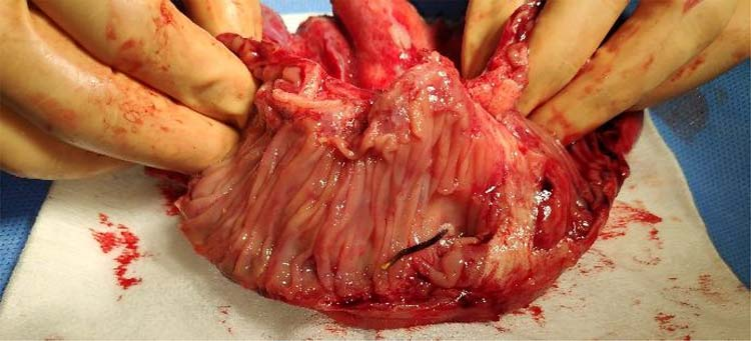
Open enterectomy specimen, in which perforation is identified in the mesenteric side of the intestinal wall by a FB.

The patient was admitted to the operating room to be submitted to exploratory laparotomy. During surgery, an incarcerated hernia whose neck measured about 3 cm was confirmed. It was possible to visualize a small bowel loop, which evidenced significant thickening of the intestinal wall and also a local abscess contained in the adjacent mesentery, without free intra-abdominal fluid. The surgical procedure was segmental enterectomy, with primary anastomosis, and also the direct correction of the hernial defect. Due to the patient’s comorbidities, surgery was performed under continuous spinal anesthesia. The operative specimen documented marked thickening of the mesentery (Fig. 4) and perforation in the mesenteric border of the loop, which was caused by a filiform FB ~3.5 mm long, compatible with a toothpick (Fig. 5).

The postoperative period was uneventful; the patient completed a 7-day cycle of piperacillin and tazobactam and was discharged on the seventh postoperative day. Histopathology evaluation of the surgical specimen documented a small intestine segment with mesenteric inflammatory infiltrate and suppuration foci related to a small area of continuity solution of the enteric wall at the mesenteric border and associated lesions of peritonitis.

Nine months after surgery, the patient was without complaints, with adequate healing, and without evidence of hernia recurrence.

## DISCUSSION

A PubMed search using the MeSH terms ‘intestinal perforation’, ‘foreign bodies’, ‘incisional hernia’ was performed. No cases of intestinal perforation within incisional hernias caused by ingested FB could be identified. Indeed, the only two case reports of intestinal perforation in incisional hernia found were caused by endoscopically placed biliary stent [6, 7].

The ingestion of FBs, although common, rarely has consequences for the patient. The main risk factor seems to be the need to use dentures or dental prostheses [2, 4] as was the case of this patient. The groups at most risk are children, elderly people, prisoners, patients with reduced visual acuity, those with alcohol abuse and those with mental illness [3, 8]. According to the literature, most of the ingested FBs are bones, fishbones and toothpicks [1, 4].

Bowel perforation caused by FB can have a broad spectrum of clinical presentation. Abdominal pain, localized or generalized, is the most common symptom [8]. The most frequent perforation sites are the terminal ileum and the rectosigmoid transition [1–4], probably due to the size of the lumen of the terminal ileum and the ileocecal valve and the angulation of the rectosigmoid junction. These conditions, which are also present in the hernia loops, may justify the perforation in the case presented. CT scan is the most sensitive and specific exam to diagnose the presence and location of intestinal perforation [9, 10]; however, the diagnosis of perforation by FBs usually is achieved intraoperatively [2, 11].

The morbidity described in FB perforations is ~35% and mortality is rare [12]. In the absence of complications, endoscopic removal is the first-line treatment, and in asymptomatic patients, vigilance may be sufficient. If complications occur, such as perforation, surgical treatment is mandatory. Surgical options may vary from primary closure of the affected orifice to segmental resection, with or without primary anastomosis, according to the location, the size of the perforation and the severity of peritonitis.

In conclusion, the ingestion of FB is quite common and rarely originates gastrointestinal perforation. Suspicion, especially in patients with risk factors, can allow for a timely diagnosis and limit the consequences for the patient.

## AUTHORS’ CONTRIBUTIONS

R.V.P. contributed to the conception of this article, review the literature and wrote the manuscript. C.F., A.M., G.G., D.M. and J.P.S. had a special contribution in the conception and the reviewing of the article.

## ETHICS STATEMENT

This manuscript is in accordance with the rules of our institutional ethics committee. Written informed consent was obtained from the patient.

## CONFLICT OF INTEREST STATEMENT

None declared.
